# Experimental parameters and infarct size in closed chest pig LAD ischemia reperfusion models; lessons learned

**DOI:** 10.1186/s12872-021-01995-7

**Published:** 2021-04-12

**Authors:** Max J. M. Silvis, Gerardus P. J. van Hout, Aernoud T. L. Fiolet, Mirthe Dekker, Lena Bosch, Martijn M. J. van Nieuwburg, Joyce Visser, Marlijn S. Jansen, Leo Timmers, Dominique P. V. de Kleijn

**Affiliations:** 1 Department of Cardiology, University Medical Center Utrecht, Utrecht, The Netherlands; 2 Department of Experimental Cardiology, University Medical Center Utrecht, Utrecht, The Netherlands; 3 Department of Cardiology, Amsterdam Cardiovascular Sciences, Amsterdam University Medical Centers, University of Amsterdam, Amsterdam, The Netherlands; 4 Department of Vascular Surgery, University Medical Center Utrecht, Heidelberglaan 100, 3508 GA Utrecht, The Netherlands; 5 Department of Cardiology, St. Antonius Hospital, Nieuwegein, The Netherlands

**Keywords:** Ischemia/reperfusion injury, Myocardial infarction, Pig model, LAD

## Abstract

**Background:**

Preclinical models that resemble the clinical setting as closely as possible are essential in translating promising therapies for the treatment of acute myocardial infarction. Closed chest pig left anterior descending coronary artery (LAD) ischemia reperfusion (I/R) models are valuable and clinically relevant. Knowledge on the influence of experimental design on infarct size (IS) in these models is a prerequisite for suitable models. To this end, we investigated the impact of several experimental features (occlusion and follow-up time and influence of area at risk (AAR)) on IS.

**Methods:**

A total of fifty-one female Landrace pigs were subjected to closed chest LAD balloon occlusion and evaluated in three substudies with varying protocols. To assess the relationship between time of occlusion and the IS, 18 pigs were subjected to 60-, 75- and 90 min of occlusion and terminated after 24 h of follow-up. Influence of prolonged follow-up on IS was studied in 18 pigs after 75 min of occlusion that were terminated at 1, 3 and 7 days. The relation between AAR and IS was studied in 28 pigs after 60 min of occlusion and 24 h of follow-up. The relation between VF, number of shocks and IS was studied in the same 28 pigs after 60 min of occlusion.

**Results:**

Increasing occlusion time resulted in an increased IS as a ratio of the AAR (IS/AAR). This ranged from 53 ± 23% after 60 min of occlusion to 88 ± 2.2% after 90 min (*P* = 0.01). Increasing follow-up, from 1 to 3 or 7 days after 75 min of occlusion did not effect IS/AAR. Increasing AAR led to a larger IS/AAR (r^2^ = 0.34, *P* = 0.002), earlier VF (r^2^ = 0.32, *P* = 0.027) and a higher number of shocks (r^2^ = 0.29, *P* = 0.004) in pigs subjected to 60 min of occlusion.

**Conclusions:**

These experiments describe the association of occlusion time, follow-up duration, AAR and VF with IS in closed chest pig LAD I/R models. These results have important implications for future I/R studies in pigs and can serve as a guideline for the selection of appropriate parameters and the optimal experimental design.

## Background

During an acute myocardial infarction (MI) due to abrupt occlusion of a coronary artery, the area of the myocardium that becomes ischemic is known as the area at risk (AAR). This AAR becomes totally infarcted when the coronary artery remains occluded. Reestablishment of coronary blood flow is the cornerstone of the current treatment for acute MI [1, 2]. Adequate and timely coronary reperfusion reduces IS, preserves cardiac tissue and function leading to a subsequent reduction of mortality [2]. The restoration of the coronary blood flow, however, can paradoxically result in additional damage to the myocardium. This phenomenon is known as ischemia/reperfusion (I/R) injury [3]. Studies in animal models suggest that it can account for up to 50% of the final IS. I/R injury is regarded an important target to preserve myocardial tissue and subsequent cardiac function [4].

Preclinical MI models are essential to develop innovative therapies against myocardial I/R injury. Rodent models are helpful to identify potential mechanisms. Direct translation from small animals to humans, however, is limited due to large anatomic, metabolic, immunologic and physiological differences. Larger animal models are therefore important for the justification of clinical trials in humans [5–7]. Pig models are among the most widely used since their coronary anatomy, physiology, metabolism, coagulation and immunologic response closely resemble those of humans [8]. Various methods of MI induction in pigs have been proposed. In general there are two main approaches. The first is an “open chest” external technique to surgically occlude the coronary artery. The second is a “closed chest” model, in which a percutaneous transluminal occlusion is performed [9]. During an open chest procedure a median sternotomy and opening of the pericardium is necessary with the disadvantage of lower epicardial temperature and surgical complications, such as infections and adhesions. The technical procedure itself could result in global ischemic preconditioning and smaller IS [9–11]. A closed chest procedure of the left anterior descending artery (LAD) balloon occlusion allows less traumatic access to the coronary arteries, more stable hemodynamic conditions and it may correspond better to the natural course of an atherothrombotic occlusion and reperfusion treatment of acute MI in humans [9, 10, 12]. Although the advantages of closed chest models are obvious, much heterogeneity exists regarding experimental design among studies, such as occlusion duration, follow-up time and the ischemic AAR [13–15]. These factors could be of influence on myocardial IS. To this extent, previous systematic assessment of historical literature has revealed several methodological determinants that could influence IS and therefore the outcome of preclinical endpoints in animal models [16]. Comparison of several of the most relevant experimental factors on IS are, however, limited.

To determine a potential effect of innovative therapies in pig models of I/R injury the choice of different experimental parameters should result in a sufficiently large IS while also sufficient remaining tissue can be salvaged from reperfusion injury (i.e. is not permanently damaged by the ischemic time). The ratio of IS and the AAR, measured with Evans Blue/2,3,5-Triphenyltetrazolium chloride (TTC) double staining, is considered the gold standard outcome measurement to investigate IS and the potential of new compounds that target lethal I/R injury [17, 18]. In dogs, this outcome is independent of the AAR [19]. In contrast, a study in pigs with an open chest method of MI showed that especially for small AAR, IS/AAR depends on the AAR [20]. Furthermore the impact of electrical defibrillation on IS in minipigs was recently investigated in an open chest model of MI. Ventricular fibrillation (VF) and defibrillation were associated with larger IS as well [21]. It remains to be elucidated whether this holds true for closed chest models.

In our ongoing studies in closed chest pig LAD ischemia reperfusion models, we noticed the impact of occlusion time, follow-up, size of the area at risk, and ventricular fibrillation on the infarct size but this was never studied in a structured manner. In the current study we therefore studied the impact of these experimental parameters on IS.

## Methods

All animal experiments were approved by the local animal welfare committee of the University Medical Center Utrecht and were executed conforming to the “Guide for the Care and Use of Laboratory Animals” and in compliance with the ARRIVE guidelines. In three substudies, we reanalyzed 51 female adult Landrace (Van Beek, Lelystad, The Netherlands) pigs (64 ± 4 kg) that were used as controls in experimental studies during 2018 and 2020.

### Premedication, anesthesia and analgesia

All pigs were pre-treated with amiodarone orally for ten days (1200 mg loading dose, 800 mg/day maintenance), clopidogrel 75 mg/day and acetylsalicylic acid (320 mg loading dose seven days before the experiment, 80 mg/day maintenance). Twenty four hours before the procedure, pigs received a buprenorphine patch (5 µg/hour). At the day of surgery, anesthesia and analgesia was induced by intramuscular (i.m.) injection of ketamine (15 mg/kg), midazolam (0.75 mg/kg) and atropine (0.015 mg/kg) followed by intravenous (i.v.) administration of thiopenthal (4 mg/kg). Pigs also received an i.v. bolus of amoxicillin/clavulanate (500/125 mg per 50 kg). Pigs were intubated and connected to a respirator with a 1:2 oxygen-air ratio. Continuous sedation and neuromuscular blockage was achieved with i.v. pancuronium (0.1 mg/kg/hour), midazolam (0.4 mg/kg/hour) and sufentanil (2.5 ug/kg/hour).

### Infarct procedure

**Fig. 1:**
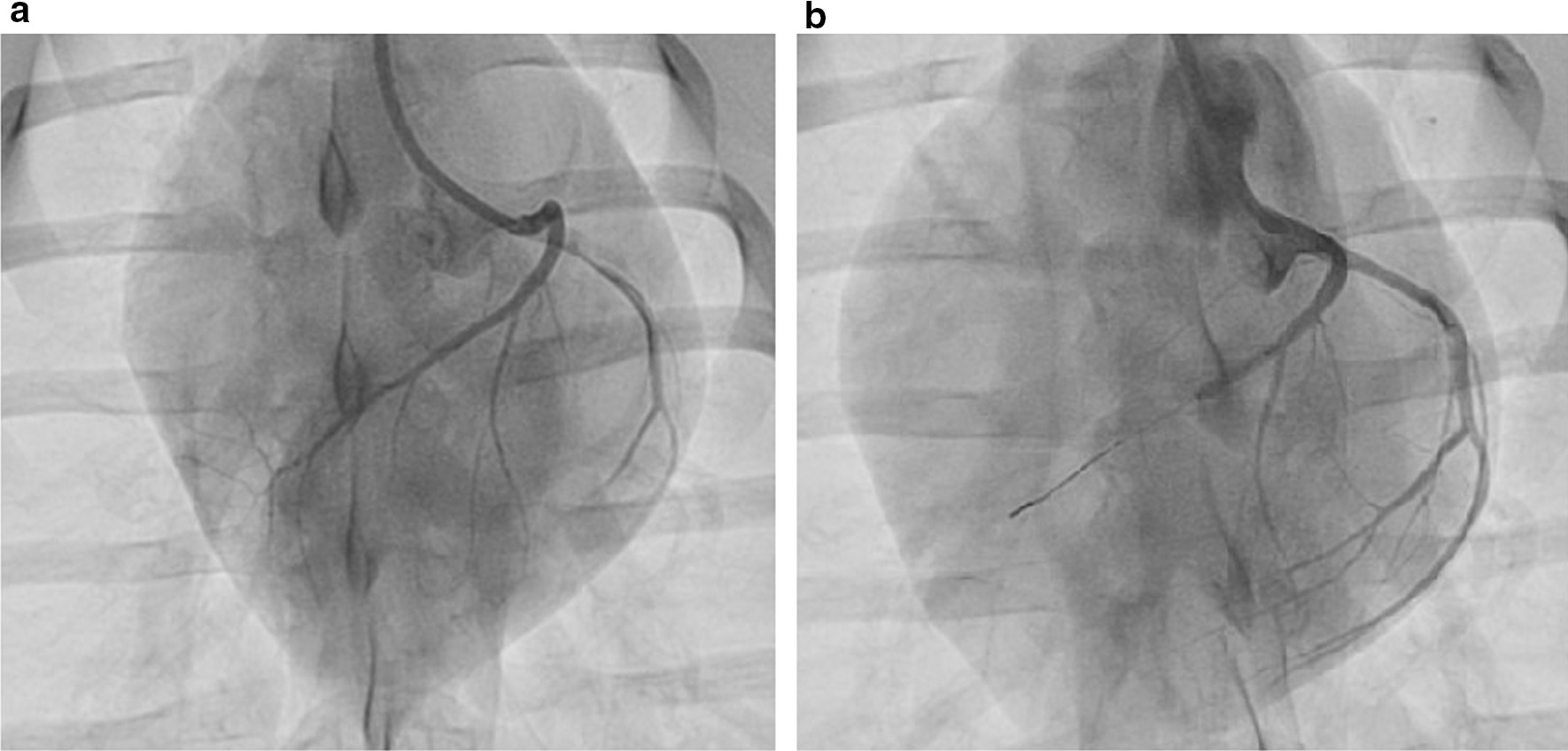
Coronary angiogram. **a** Prior to occlusion, **b** Successful LAD coronary artery balloon occlusion

Pigs were subjected to closed-chest LAD coronary artery balloon occlusion. After arterial accesses was obtained, a catheter (8FR JL4 guiding) was placed in the left coronary tree. After a successful coronary angiogram the diameter was measured after an i.v. bolus of nitroglycerin and an adequately sized (2.5–3.5 mm) balloon (1:1 balloon to vessel ratio) was placed mid LAD, preferably directly after the first diagonal and occlusion was confirmed (Fig. 1). The size of the AAR is the result of two methodological determinants: 1. Individual coronary anatomy and 2. Location of the balloon. When the first diagonal is very small, we have the experience that there is high risk of refractory VF and mortality and in that case we occlude below the second diagonal. Of note, when we occlude before the first diagonal almost all pigs develop refractory VF and die during infarct induction, therefore occlusion should at least be below the first diagonal. A coronary angiogram was repeated after 30 min of occlusion to confirm complete occlusion of the LAD. In case of sustained ventricular tachycardia or VF, pigs were defibrillated (200 J) and received an additional i.v. bolus of 150 mg amiodarone (with a maximum of 3 bolus). Prior to reperfusion, a coronary angiogram was performed to confirm successful occlusion throughout the whole period of time. After reperfusion and observation for approximately 2–3 h, the surgical wound was closed and animals were weaned from anesthesia.

### Protocols

**Fig. 2:**
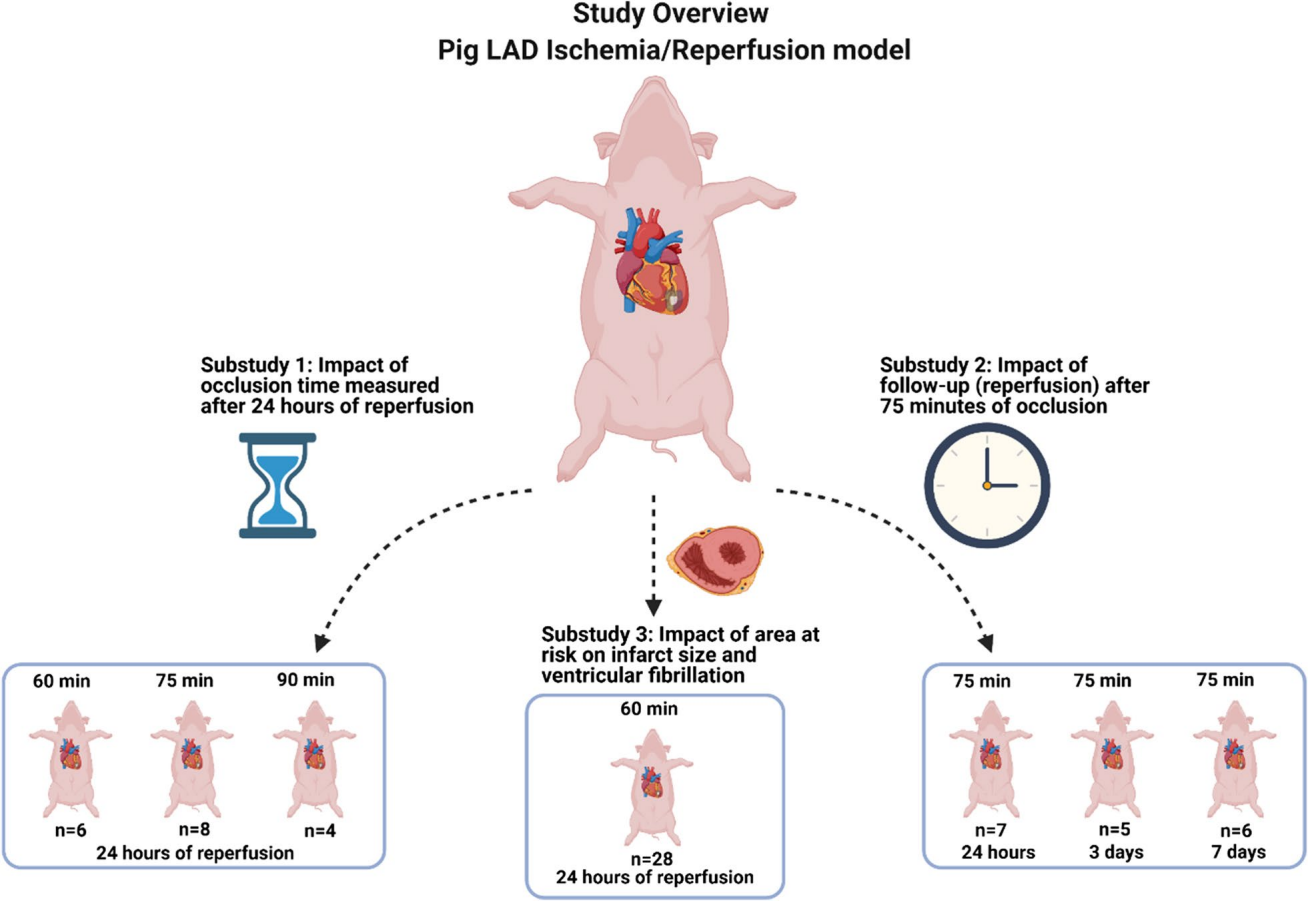
Study overview

A detailed description of the different protocols can be found in Fig. 2.

*Substudy 1 (n* = *18)*: Impact of occlusion time.

Pigs were subjected to three different occlusion times, 60-, 75-, and 90-min and after 24 h of follow-up (reperfusion) pigs were terminated and infarct size was measured.

*Substudy 2 (n* = *18)*: Impact of follow-up (reperfusion).

Pigs were subjected to 75-min of occlusion and after one day, three days, or seven days of follow-up (reperfusion) pigs were terminated and infarct size was measured.

*Substudy 3 (n* = *28)*: Impact of area at risk on infarct size and VF.

Pigs were subjected to 60 min of occlusion and after 24 h of follow-up (reperfusion) pigs were terminated and infarct size was measured.

### Area at risk and infarct size measurement

Before the heart was excised under general anesthesia as previously described, the LAD was occluded at the same site as during infarct induction. After verification of complete vessel occlusion, Evans Blue solution (1.00 g was dissolved in 50 ml of 0.9% NaCl) was infused in the left (30 ml of the solution) and right coronary (20 ml of the solution) arteries in order to assess the AAR. Pigs were then sacrificed by exsanguination. The heart was excised and the left ventricle (LV) was cut in five slices. The slices were incubated in 1% TTC (Sigma-Aldricht Chemicals, Zwijndrecht, The Netherlands) in 37 degrees Celsius 0.9% NaCL for 15 min to discriminate between infarct tissue and viable myocardium (AAR that was not infarcted). Each slice was weighed and photographed at the basal and apical side. Images were analyzed with ImageJ software (NIH, Bethesda, MD, USA). AAR was calculated as a percentage of the LV. IS was calculated as a percentage of the AAR.

### Statistical analysis

Data are expressed as mean ± standard deviation unless stated otherwise. IS was compared using a one-way Analysis of Variance (ANOVA) or Kruskal Wallis when non-normally distributed data, followed by appropriate post hoc tests. The linear relationship of two continuous parameters was assessed using the Spearman’s rank correlation coefficient and simple linear regression. All data were analyzed using SPSS Statistics 25.0 (IBM Corp, Armonk, NY, USA). A two-sided *P*-value of < 0.05 was regarded statistically significant.

## Results

### Duration of ischemia and IS in closed chest LAD I/R model

**Fig. 3:**
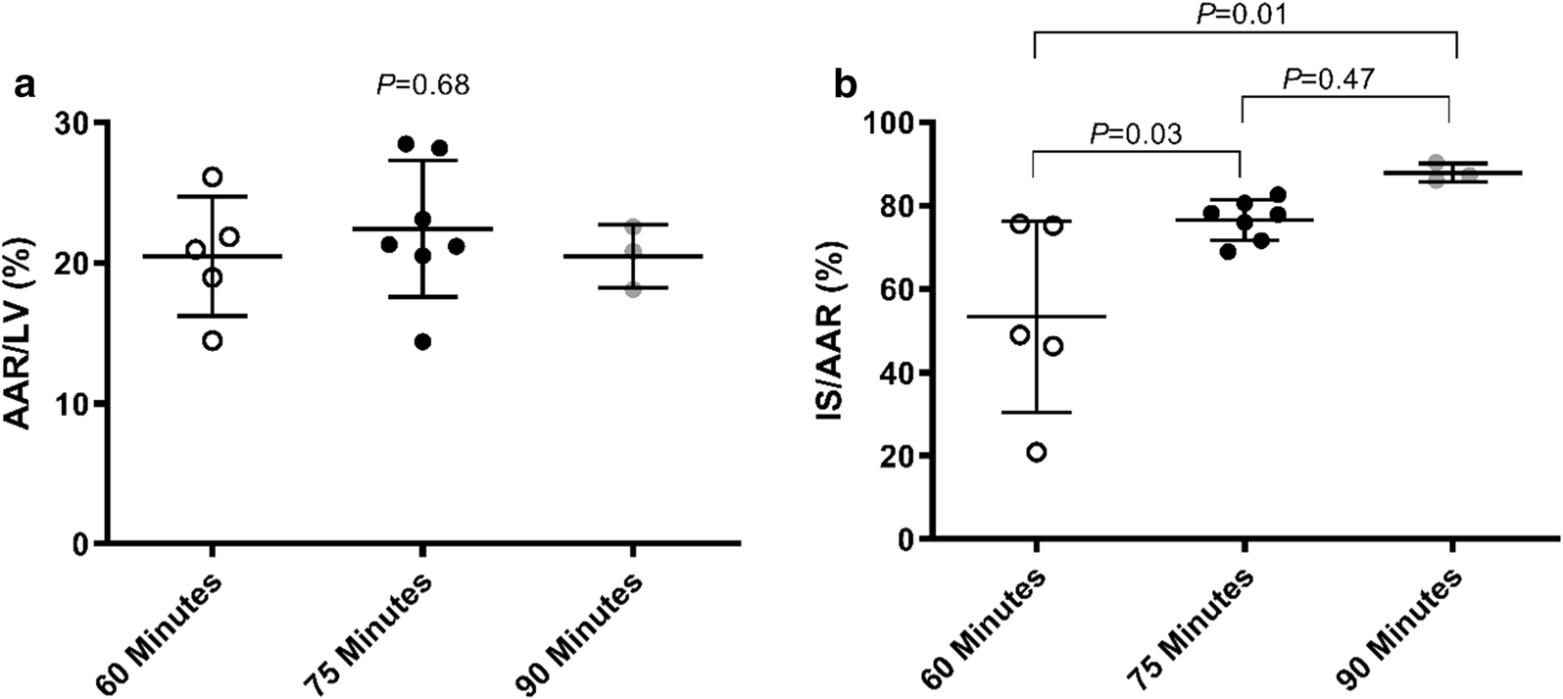
Occlusion time and infarct size after 24 h of reperfusion. **a** Shows similar AAR in the three groups. **b** The IS as a percentage of the AAR was higher in the pigs with longer occlusion time

For head to head comparison of the influence of occlusion time, closed chest LAD occlusion was performed in a total of eighteen pigs with varying occlusion times of 60 min (n = 6), 75 min (n = 8) and 90 min (n = 4). In each group one pig died during infarct induction due to refractory VF leaving fifteen for final analysis ((60 min (n = 5), 75 min (n = 7), 90 min (n = 3)). Myocardial IS was determined in the remaining fifteen pigs 24 h after reperfusion. There was no difference in the AAR as a percentage of the LV between the three occlusion groups (60 min group 20.48 ± 4.2%, 75 min group 22.45 ± 4.9%, 90 min group 20.50 ± 2.2%, *P* = 0.68) (Fig. 3a).

**Fig. 4:**
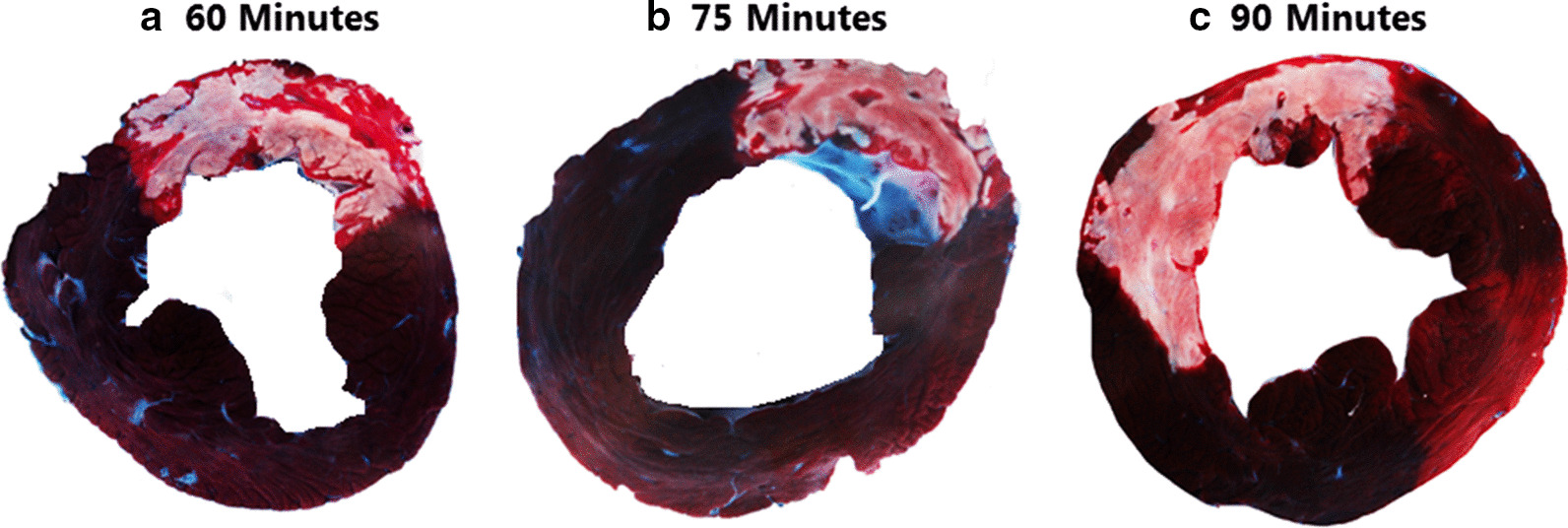
Representative pictures of myocardial slices of the three different occlusion times. The dark area represents the remote area, the area at risk is stained red and the infarcted myocardium is stained white/pale. **a** 60 min of occlusion. **b** 75 min of occlusion. **c** Almost complete transmural infarction after 90 min of occlusion

With an increasing occlusion time the IS as a percentage of the AAR (IS/AAR) is increased (60 min group 53 ± 22.9%, 75 min group 76.57 ± 4.8%, 90 min group 88 ± 2.2%) (*P* = 0.01) (Fig. 3b). The standard deviation of the 60 min group (± 22.9%) was larger than the 75 min and 90 min group (± 4.8%, ± 2.2% respectively). Figure 4 shows representative pictures of myocardial slices of the three different occlusion times.

### Follow up and IS

**Fig. 5.**
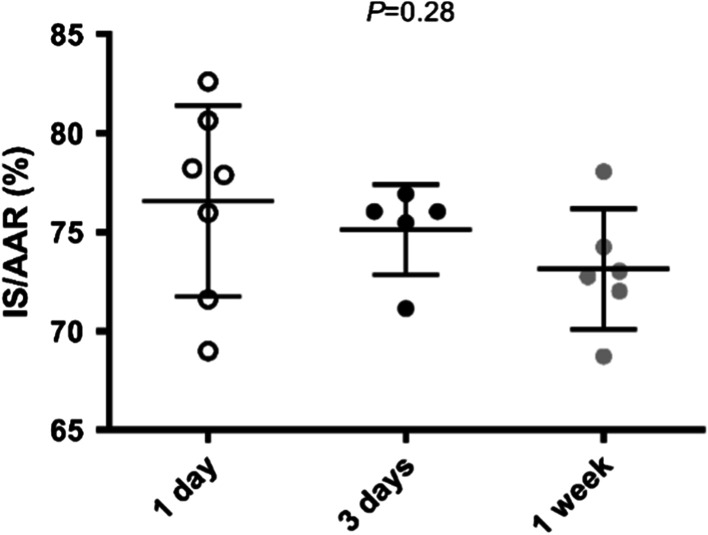
75 min of occlusion with different follow-up. Prolonged follow-up duration did not result in differences in IS/AAR

Myocardial IS was measured in 7 pigs that were terminated after one day, 5 pigs after three days and 6 pigs after one week. There were no significant differences in IS/AAR between the pigs. (one day; 76.7 ± 4.8% vs. three days; 75,1 ± 2.3% vs. one week; 73,1 ± 3.0%, *P* = 0.28) (Fig. 5).

### AAR and IS after 60 min of LAD occlusion

**Fig. 6:**
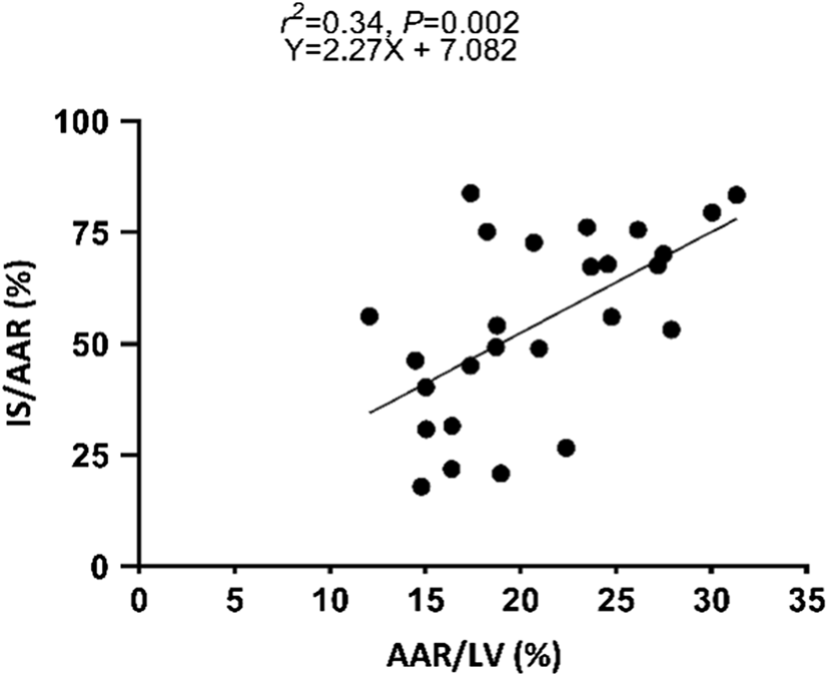
Correlation between AAR and IS/AAR. Scatterplot showing a significant correlation between the size of the AAR and IS as a percentage of AAR in the 60 min group (n = 26)

A total of twenty-eight pigs were subjected to 60-min of transluminal mid-LAD occlusion and AAR and IS were measured after 24 h of follow-up. Two pigs died during infarct induction due to refractory VF, allowing analysis of twenty-six pigs with 60 min occlusion time. Mean AAR was 20.9 ± 5.3%. Mean IS/AAR was 54.6 ± 20.6% and thus confirms the results for 60 min of occlusion as observed in substudy 1. AAR/LV correlated significantly with IS/AAR (*r*^2^ = 0.34, *P* = 0.0016) (Fig. 6).

### Time to defibrillation and number of shocks

**Fig. 7:**
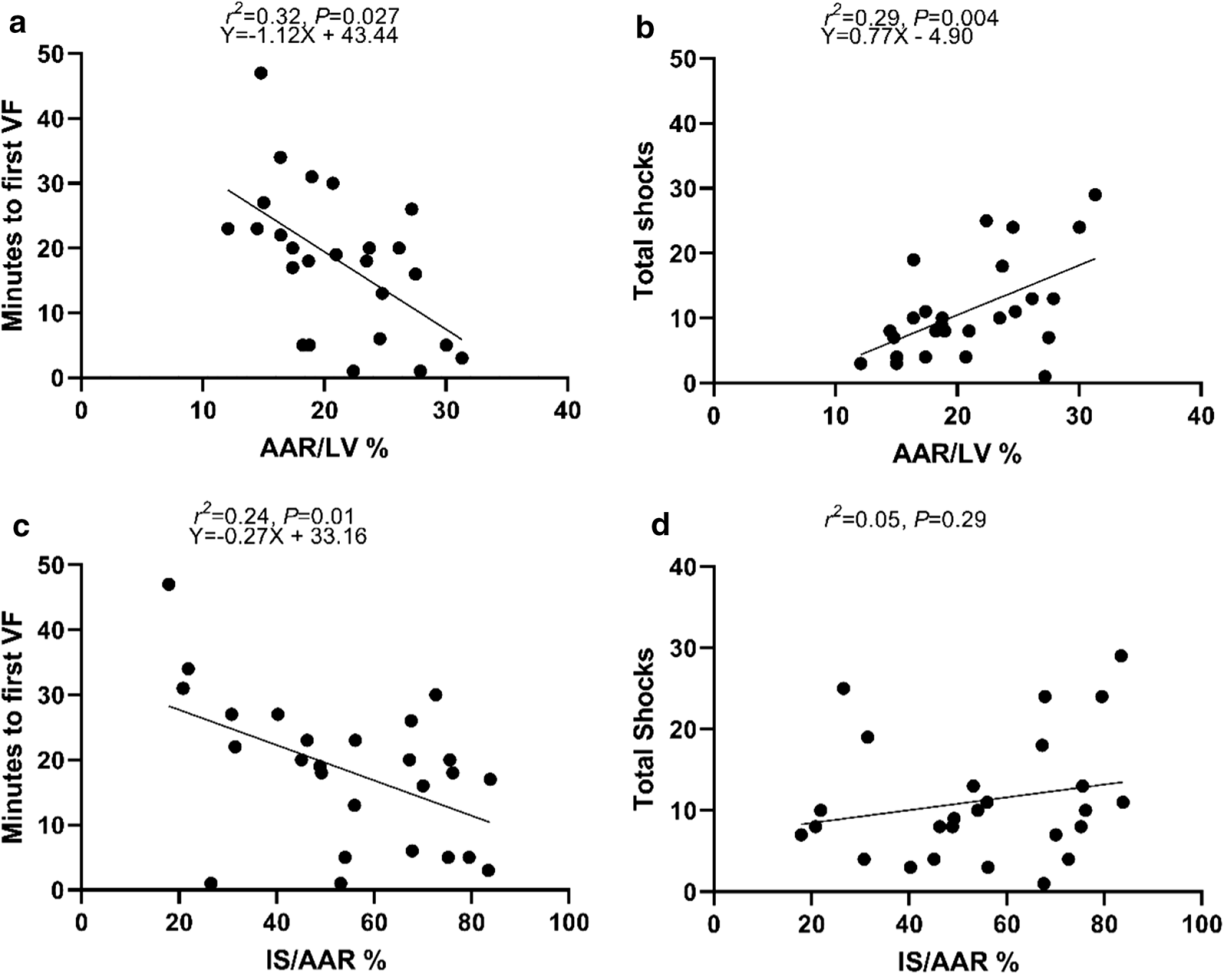
Correlation between AAR/LV, IS/AAR and VF/defibrillation. **a**, **b** Scatterplots showing a significant correlation between the size of the AAR and time to VF and the number of shocks. **c**, **d** IS/AAR shows a significant correlation with time to VF but not with the total number of shocks

Data on time to defibrillation and number of shocks were available in the same 26 pigs that were subjected to 60 min of occlusion. The incidence of VF was 100%. The mean time from the start of occlusion to the onset of a first episode of VF was 18 ± 11 min and this, and other episodes of VF during occlusion, were terminated by defibrillation (200 J) immediately. The mean number of defibrillations was 12 ± 7.5. AAR/LV shows a significant negative correlation with minutes to first episode of VF (*r*^2^ = 0.32,* P* = 0.027) (Fig. 7a). AAR/LV also showed a positive correlation with the number of shocks required (*r*^2^ = 0.29,* P* = 0.004) (Fig. 7b). IS/AAR showed a negative correlation with time to first episode of VF (*r*^2^ = 0.24,* P* = 0.01) (Fig. 7c). IS/AAR did not correlate with number of shocks (*r*^2^ = 0.05,* P* = 0.29) (Fig. 7d).

## Discussion

Appropriate experimental parameters in a pig model of MI are pivotal when investigating the potential of cardio protective treatments that aim to reduce IS. In the present study, we describe the association between several parameters: occlusion time, duration of follow-up, size of AAR, time to VF and number of shocks and myocardial IS in the closed chest LAD pig model of I/R injury.

### Duration of ischemia

Our results in a closed chest pig LAD I/R model show the impact of increasing occlusion time on IS and are in line with previously published studies in open chest pig models, which also showed almost complete transmural infarction after 90 min of ischemia [22, 23]. Myocardial IS is the result of ischemia and I/R injury. Larger ischemic injury results in less myocardial tissue that can be potentially injured by reperfusion. The proportion of (lethal) reperfusion injury in a pig with 90 min of LAD occlusion will therefore be very low or almost absent. A shorter occlusion time, such as 60 min, results in a smaller IS/AAR and subsequently more myocardial tissue that can be injured by reperfusion. This, however, comes with a larger variation in IS/AAR, requiring a larger sample size to detect potential differences in treatment and control groups [14, 24].

### Follow-up

The follow-up duration was not statistically significant associated with IS/AAR in pigs subjected to 75 min of LAD occlusion. Data on the impact of increasing follow-up duration after I/R injury in pig models of MI is scarce but a historic report in an open chest mouse model of myocardial I/R injury showed that the IS was increasing when the follow-up duration was increased from 120 to 240 min [25]. This was only seen in the model with 30 or 45 min of LAD occlusion. IS in mice was not increased with increasing reperfusion time when occlusion was longer than 60 min [25]. Cardiac magnetic resonance (CMR) imaging in pigs (40 kg) following 40 min of myocardial ischemia showed a dynamic distribution of post-MI edema that has a clear impact on CMR-measured AAR, with an overestimation at 2 h of reperfusion and an underestimation at 24 h compared to the AAR that was measured during occlusion[26, 27]. After four and seven days after the start of reperfusion, the AAR was similar to the values measured during occlusion. This seems to be in contrast with our findings, however, their AAR and IS calculations with CMR were not compared with Evans Blue/TTC staining. Furthermore, the so called “first wave of edema” at 2 h of reperfusion was not investigated in our study. Importantly myocardial ischemia was much shorter (40 min) while our occlusion time was 75 min. Although CMR has the advantage that serial AAR and IS can be determined in one pig on multiple timepoints, it has the disadvantage that accurate determination of the AAR is difficult. For future studies, it would be valuable to compare CMR measurements of AAR and IS with Evans Blue/TTC staining on the explanted heart.

In conclusion, our results show that IS/AAR does not increase after one day. This indicates that for the investigation of I/R injury a follow-up duration of 24 h is sufficient and leads to final IS and does not provide evidence of an increasing infarct due to I/R injury in the following days. However, our results are limited to pigs subjected to 75 min of occlusion.

### AAR

We observed that in the 60 min occlusion model AAR/LV correlated with the IS/AAR. Data on the relation between AAR/LV and IS/AAR in pigs is scarce but one previous study observed that especially for small AAR, IS/AAR depends on AAR and therefore suggests that the AAR should ideally be between 20 and 30% of the LV [20, 28]. Our results underline that it is essential that IS is reported as a percentage of the AAR and not only as a percentage of the LV since effects of an experimental treatment can be masked by variations in AAR.

### VF and defibrillation

AAR/LV and IS/AAR were negatively correlated with time to VF and required defibrillation. The correlation between IS/AAR and time to VF, however, could be driven by a larger AAR which also correlated with IS/AAR. Defibrillation showed to be an additional determinant of IS as end point of myocardial I/R injury in an open chest model [21]. In our model, all pigs developed VF after occlusion. We were not able to investigate if defibrillation itself is associated with a larger IS. Our findings indicate that the incidence of VF and the number of defibrillations must be reported since it is associated with AAR and IS size. Furthermore we did not observe an association between increasing number of shocks and IS. Electrical injury is therefore unlikely to be an independent covariate. This is also in line with previous results [21].

### Translational challenges

Even with a suitable pig model to investigate the influence of I/R injury it is important to keep in mind that there are major differences with regards to the humans we eventually aim to treat. For example, the balloon occlusion in healthy pig coronary arteries differs from the diseased atherosclerotic coronary arteries in humans. Additionally, development of MI in patients is much slower than in pigs due to many factors, such as ischemic precondition, the occurrence of stuttering occlusion/reperfusion, and collateral blood flow. In the current study we show that over 50% of the AAR is infarcted after 60 min whereas previous reports show that this takes about 4.5 h in humans [16]. Furthermore, in humans even after 12 h of acute MI myocardial tissue can still be salvaged [29]. Additionally, the actual proportion of reperfusion injury in final IS in patients remains unclear and is probably heavily influenced by other factors, such as comorbidities and comedication [30]. Future studies should therefore also focus on the mechanisms by which I/R injury plays a role in patients.

### Limitations

Several limitations are associated with the present study. First, our results have an exploratory purpose and should be interpreted with caution as the limited sample size increases the risk for type 2 errors. Second, we used female adult Landrace pigs, thus results may differ from other strains, males and younger pigs. The influence of follow-up duration was only investigated in the pig model with 75 min of occlusion and cannot be applied to pigs subjected to different occlusion times. The same statement holds true for the relation between AAR, VF and defibrillation, with IS which was only investigated after 60 min of occlusion.

## Conclusion

This study demonstrates the impact of experimental design on IS in closed chest LAD pig models of MI. These results have important implications for future I/R studies in pigs and facilitate the selection of the appropriate parameters for the optimal experimental read-out.

## Data Availability

The datasets used and/or analyzed during the current study are available from the corresponding author on reasonable request.

## Abbreviations

AAR: Area at risk
ANOVA: One-way Analysis of Variance
CMR: Cardiac Magnetic Resonance
I/R: Ischemia reperfusion
IS: Infarct size
LAD: Left anterior descending coronary artery
LV: Left ventricle
MI: Myocardial infarction
SPSS: Statistical Package for the Social Sciences
TTC: 2,3,5-Triphenyltetrazolium chloride
VF: Ventricular fibrillation
